# PP97 Conduction System Pacing Implantation Through Electroanatomic Mapping-Guided Versus Fluoroscopy In Patients With Severe Bradyarrhythmias

**DOI:** 10.1017/S0266462324002617

**Published:** 2025-01-07

**Authors:** Diego Infante-Ventura, Beatriz León-salas, Aránzazu Hernández-Yumar, Renata Linertová, Javier García García, Raúl Quirós López, Estefania Herrera Ramos, Mar Trujillo-Martín

## Abstract

**Introduction:**

Electroanatomic mapping (EAM) has been shown to be an alternative procedure to fluoroscopy for the implantation of conduction system pacing (His-bundle pacing [HBP] and left bundle branch pacing [LBBP]) in patients with severe bradyarrhythmias, mainly those vulnerable to ionizing radiation. However, the evidence of its beneficial and harmful effects has not been assessed in a systematic review (SR).

**Methods:**

An SR of the available scientific literature was conducted on the safety, effectiveness, and cost-effectiveness of implantation of the HBP and LBBP using EAM system versus fluoroscopy in patients with bradycardia with an indication for pacing therapy. Cochrane methodology and PRISMA statement for reporting were followed. A partial economic evaluation was carried out to compare the costs of both pacemaker implantation strategies from the perspective of the Spanish National Health System. A budget impact analysis was also conducted with a five-year horizon.

**Results:**

Seven comparative observational studies (N=259) analyzing the use of EAM versus fluoroscopy were selected. Statistically significant differences were observed in total fluoroscopy time: −9.87 minutes (95% confidence interval [CI]: −14.20, −5.53; p<0.01; I2=95%; k=7; n=231); His-lead fluoroscopic time: −8.08 minutes (95% CI: −10.36, −5.81; p<0.01; I2=0%; k=2; n=50); and His-lead radiation dose: −17.21 mGy (95% CI: −24.08, −10.34; p<0.01; k=1; n=20). No differences in total radiation dose, procedural success, immediate procedure-related complications, electrode revision, or device infection were found. The use of EAM represents an increase of EUR1,397.81 (USD1,513.88) per patient and a net budget impact of EUR1.63 million (USD1.77 million).

**Conclusions:**

No differences between EAM and fluoroscopy in terms of procedure success and safety were found. Therefore, EAM is a valuable alternative for patients who should not be exposed to ionizing radiation. The inclusion of EAM systems, for the indication under study, in routine clinical practice would mean an increase in costs for the Spanish National Health System.

